# Environmental DNA (eDNA) Metabarcoding in the Fish Market and Nearby Seafood Restaurants in Taiwan Reveals the Underestimation of Fish Species Diversity in Seafood

**DOI:** 10.3390/biology10111132

**Published:** 2021-11-04

**Authors:** Hung-Tai Lee, Cheng-Hsin Liao, Te-Hua Hsu

**Affiliations:** 1 Department of Environmental Biology and Fisheries Science, National Taiwan Ocean University, Keelung 20224, Taiwan; htlee@ntou.edu.tw (H.-T.L.); chliao@mail.ntou.edu.tw (C.-H.L.); 2 Department of Aquaculture, National Taiwan Ocean University, Keelung 20224, Taiwan; 3 Center of Excellence for the Oceans, National Taiwan Ocean University, Keelung 20224, Taiwan

**Keywords:** biodiversity, DNA barcoding, fisheries, aquaculture

## Abstract

**Simple Summary:**

Seafood, especially the traditional one in Taiwan, is rarely sourced from a fixed species and routinely from similar species depending on their availability. Hence, species diversity in seafood could be potentially complicated. While a DNA-based approach has been extensively utilized for species identification, a large scale of seafood species identification in fish markets and nearby seafood restaurants could be challenging (e.g., elevated cost and time-consuming only for a limited number of species identification). Environmental DNA (eDNA) metabarcoding has emerged as a promising tool for the simultaneous identification of multiple species in the environments. In this work, we aimed to identify the majority of fish species potentially consumed in fish markets and nearby seafood restaurants using this novel approach. A total of 153 fish species have been identified. Specifically, 22 chondrichthyan fish, 14 Anguilliformes species, and 15 Serranidae species were potentially linked with smoked sharks, braised moray eels, and grouper fish soups, respectively. This is the first study to examine the feasibility of a large scale of seafood identification using eDNA metabarcoding. Our findings also further imply the species diversity in traditional seafood might be seriously underestimated and crucial for the conservation and management of marine resources.

**Abstract:**

Seafood, especially the traditional one in Taiwan, is rarely sourced from a fixed species and routinely from similar species depending on their availability. Hence, the species composition of seafood can be complicated. While a DNA-based approach has been routinely utilized for species identification, a large scale of seafood identification in fish markets and restaurants could be challenging (e.g., elevated cost and time-consuming only for a limited number of species identification). In the present study, we aimed to identify the majority of fish species potentially consumed in fish markets and nearby seafood restaurants using environmental DNA (eDNA) metabarcoding. Four eDNA samplings from a local fish market and nearby seafood restaurants were conducted using Sterivex cartridges. Nineteen universal primers previously validated for fish species identification were utilized to amplify the fragments of mitochondrial DNA (12S, COI, ND5) of species in eDNA samples and sequenced with NovaSeq 6000 sequencing. A total of 153 fish species have been identified based on 417 fish related operational taxonomic units (OTUs) generated from 50,534,995 reads. Principal Coordinate Analysis (PCoA) further showed the differences in fish species between the sampling times and sampling sites. Of these fish species, 22 chondrichthyan fish, 14 Anguilliformes species, and 15 Serranidae species were respectively associated with smoked sharks, braised moray eels, and grouper fish soups. To our best knowledge, this work represents the first study to demonstrate the feasibility of a large scale of seafood identification using eDNA metabarcoding approach. Our findings also imply the species diversity in traditional seafood might be seriously underestimated and crucial for the conservation and management of marine resources.

## 1. Introduction

Seafood generally refers to a diverse range of aquatic organisms utilized for food and has been one of the major traded food commodities in the world [1]. Globally, there is a growing demand for seafood since human consumption continues to increase annually by 3.1% [2]. Over the past few decades, a rapid growth in aquaculture production has certainly enabled a substantial increase in the supply of seafood and is projected to outnumber capture fisheries production by 2030 [3]. However, there is still a considerable amount of seafood from capture fisheries [2,3]. Furthermore, the species diversity of seafood from capture fisheries could be more complex due to the nature of aquatic biodiversity.

Seafood identification has increasingly received attention since seafood mislabeling has been reported, raising public awareness of the safety and sustainability of seafood [4]. Moreover, seafood mislabeling could lead to seafood fraud when a low commercial value seafood is intentionally labeled as a high commercial value one. In general, mislabeling of seafood could be attributable to the inaccurate identity and origin of aquatic organisms used for seafood [5]. Misidentification of seafood species is perhaps one of the most common problem for seafood mislabeling. To address this issue, it requires accurate and reliable methods for the identification of seafood species.

Conventionally, morphological characters are routinely used for the taxonomical identification of seafood species [6]. While visual discrimination of seafood species is certainly simple and cheap, it also requires experts with well-trained experience. Moreover, visual discrimination of seafood species could be difficult or impossible since morphological characters are often removed, altered, or destroyed following the process, storage, and transport of seafood [6]. Alternatively, DNA-based methods have proven to be effective for accurate seafood species identification that have been frequently used for the authentication of seafood products [7,8,9,10,11,12]. However, conventional DNA-based approach often requires DNA samples from seafood products, and it could be challenging when specimens are precious and/or required to keep alive. Moreover, a large scale of DNA-based seafood species identification also requires a considerable number of DNA samples. In this case, it could be laborious and expensive for species identification using conventional DNA-based approach.

Environmental DNA (eDNA) is generally considered as DNA present in a variety of environmental samples, including soil, air, or water [13,14]. Recently, eDNA-based sampling has emerged as a promising tool to monitor the presence of species within an environment. With the aid of high throughput sequencing technology, eDNA metabarcoding has further allowed the simultaneous identification of multiple species present in environments [15,16]. Hence, eDNA sampling has been extensively applied to explore the biodiversity, distribution, and habitat of aquatic organisms [15,17,18,19,20].

Taiwan is a relatively small island (36,197 km^2^) with rich marine biodiversity and resources since it is geographically located in the Western Indo-Pacific region, a hotspot of marine biodiversity [21]. More than 3000 finfish species have been recorded in the water of Taiwan, accounting for 9% of the fish species in the world [22]. Coastal fisheries in Taiwan are highly active with more than 20 fishery sectors in this region [23]. Consequently, seafood has been one of major food resources in Taiwan. However, seafood in Taiwan, especially in fish markets and seafood restaurants, is rarely sourced from a fixed species and routinely prepared with similar species depending on their availability. Moreover, the oversimple and inconsistent names of seafood also raise the concerns on the exact identities of seafood. Hence, the species diversity of seafood could be potentially complicated in Taiwan.

In the present study, we aim to determine whether it is feasible to identify fish species potentially consumed in fish markets and seafood restaurants using eDNA metabarcoding. Unlike the conventional DNA-based approach, we have collected and analyzed eDNA samples in a local fish market and nearby seafood restaurants to provide a reference list of fish species potentially consumed, particularly those threatened, commercially important, nutrition valued species. Our findings are also expected to provide a comprehensive understanding of the species diversity in seafood for the conservation and management of marine resources.

## 2. Materials and Methods

### 2.1. Sample Collection

**Figure 1:**
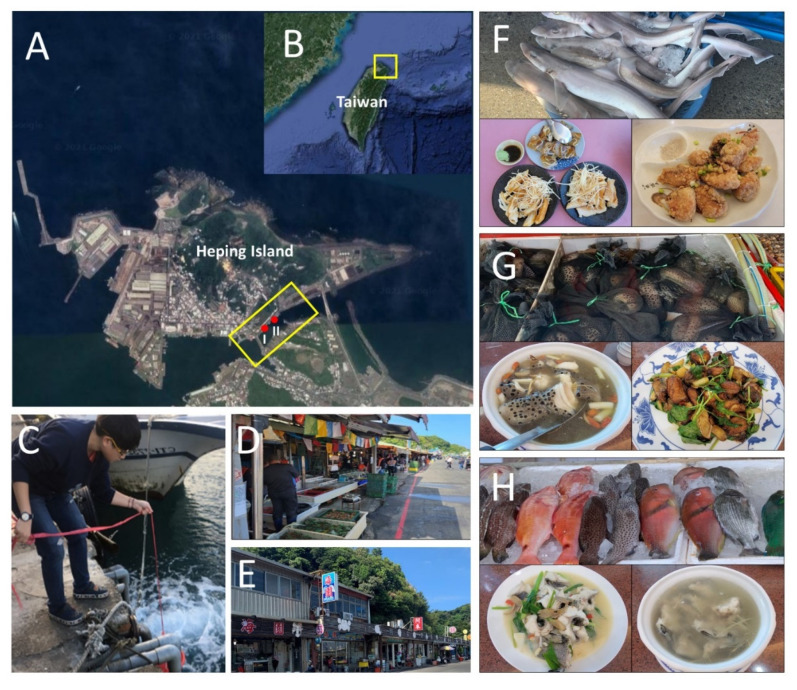
eDNA sampling site information: eDNA samplings were performed in the Heping Island of Keelung city in the northeastern Taiwan (**A**,**B**). Water samples were collected in two sampling sites (**C**) near the drains of the fish market (**D**) and its nearby seafood restaurants (**E**). Traditional seafood commonly found in sampling sites, including smoked shark (**F**), braised moray eel and Anguilliformes species (**G**), grouper fish soups and Serranidae species (**H**). The yellow rectangle indicates the region of the fish market and its nearby seafood restaurants. The red circles mark the location of two sampling sites (I, II).

This research was conducted in the Heping Island fish market in Keelung City, the northeastern Taiwan from December 2020 to January 2021 (Figure 1A–C). Heping Island fish market is a small market (area: 200 m × 10 m), contains 20-30 traditional seafood stalls and restaurants (Figure 1D,E). The seafood here includes live fish, fresh fish, and processed products. It is mainly caught in the wild and comes from nearby fish harbors. Most seafood can be observed directly in the fish tank and at the food stall (Figure 1F–H). In order to reflect seafood diversity, two sampling sites were chosen near the drain of the fish market (100 m between two sites) (Figure 1A,D,E). We sampled twice in total, with an interval of about 1 month. We extracted the seawater near the drain with a bucket, and then 1 L seawater was filtered immediately through a Sterivex cartridge (0.45 μm, Millipore SVHV010RS, Merck Millipore, Billerica, MA, USA) with a syringe (500 mL) by hand. Each sampling was taken three times, and the interval was 10 min. Filtered samples were placed on ice until they were taken back to the laboratory.

### 2.2. DNA Extraction

DNA extraction was conducted as previously described [24] with some modifications. A lysis buffer mix (PBS 220 μL, Buffer AL 200 μL, Proteinase K 20 μL; 440 μL total volume) was introduced to the cartridge through the inlet. Both ends of the cartridge were sealed by Luer-Lock stoppers and the cartridge was incubated at 56 °C for 30 min with mild rotation. After the incubation, Luer-Lock was removed from the Sterivex cartridge, and the A lysis buffer mix was pipetted from the inlet. Mix sample (200 μL) was further purified by Qiagen DNeasy Blood and Tissue Kit (ThermoFisher Scientific, Waltham, MA, USA) according to the manufacturer’s protocol. DNA concentration of each sample was checked by using the Qubit dsDNA HS Assay (Invitrogen, Carlsbad, CA, USA)

### 2.3. DNA Library Preparation

Amplified gene fragments were prepared for NovaSeq 6000 sequencing according to the Illumina Sequencing Library preparation guidelines (Illumina, Inc., San Diego, Califor-nia, USA). Three mitochondrial gene regions were amplified by 19 universal primers, including: 12S rRNA (MiFish-U/E), ND5 (MiFish-tuna-ND5), and COI (Fish-miniA/C/E; Fish1/2; Fish1F/2F with Shark COI-MINIR, Shark-MiniV1-R, and Shark-MiniV2-R) as described [25,26,27,28] with modification (Table 1).

Each 20 µL PCR reaction contained 10 µL of 2x PCRBIO HS Taq Mix, 1 µL of each primer (10 µM), 7 µL ddH2O, and 1 µL of DNA extract. The following cycling conditions were used: 5 min at 95 °C (1×); 1 min at 95 °C, 30 s at 48 °C, and 45 s at 72 °C (38×); 5 min at 72 °C (1×). Three PCR replicates were amplified from each sample and then pooled for a single PCR cleanup with the QIAquick 96 PCR purification kit (Qiagen; 60 µL elution volume). Agarose (2% *w*/*v*) gel electrophoresis was used to verify the amplification of samples. PCR products were pooled and quantified using Qubit dsDNA HS Assay before preparation for the library. The library was following the protocol of the Illumina DNA PCR-Free Library Prep. The library was sequenced with a 300-cycle S4 kit on the NovaSeq 6000 (with paired-end 150-bp reads, PE150) following the NovaSeq XP workflow. Library preparation, sequencing, and base calling were carried out by Genomics BioSci & Tech (http://www.genomics.com.tw/ accessed on 1 February 2021).

### 2.4. Data Process

The overall quality of the Novaseq reads was inspected by FastQC [29]. Adaptor sequence and low-quality tails in raw sequence data were trimmed (quality ≤ 10) by Trimmomatic 0.32. Sequencing reads were filtered to remove reads shorter than 150 bp. The remaining reads were merged using BBMerge algorithm with default parameter settings and reads [30]. The assembled reads were further demultiplexed by different primer sets (12S rRNA: MiFish-U/E; ND5: MiFish-tuna-ND5; COI: Fish-miniA/C/E, Fish1/2, Fish1F/2F with Shark COI-MINIR, Shark-MiniV1-R, and Shark-MiniV2-R) by Cutadapt 3.4 [31]. In order to remove reads with either ambiguous sites (Ns) or those showing unusual and too short lengths with reference to the expected size of the PCR amplicons. Primer clipping and lengths control of reads were also used Cutadapt 3.4 [31].

### 2.5. Taxonomic Assignment

The pre-processed reads from the above pipeline were further dereplicated by using a ‘derep_fulllengthzrusing’ command in VSEARCH [32]. Keep only sequences with an abundance equal to or greater than 2. OTU clustering and chimera detection in derep-licated reads for each primer set was used “-cluster_otus” command in USEARCH by default setting. All OTUs were subjected to local BLASTN searches against a fish mitogenome database MitoFish V3.68 [33,34]. The top BLAST hit with a sequence identity of more than or equal to 97% and sequences larger than 100 bp was applied to species assignments of each OTU. Taxonomic assignment to the species level, and all the scientific names were checked to remove the synonyms by NomenMatch (http://match.taibif.tw/ accessed on 10 August 2021). The processed OTUs from all primer sets were built to the OTU table. The OTU occurrences for each sample were performed “usearch_global” with a 97% similarity threshold command in VSEARCH [32].

### 2.6. Statistical Analysis

For the analysis of the species diversity, the species data were analyzed with a presence/absence approach [18]. The species matrix was obtained by the Jaccard similarity index using the excel VBA. Principal Coordinates Analysis (PCoA) was then used to investigate the relationship between samples generated through the species matrix by GenAlEx 6.503 [35].

## 3. Results

### 3.1. Identification and Classification of Fish Species from eDNA Samplings

A totoal of 50,534,995 reads of three mitochondrial genes (12S, ND5, COI) were obtained from 4 eDNA samplings, including 6,995,762 reads for December-I, 10,194,137 reads for December-II, 22,323,200 reads for January-I, and 11,021,896 reads for January-II (Table 2). Following the local BLASTN searches, 417 OTU related to fish speics were obtained. A total of 153 fish species with high sequence similarity (above 0.97) were retained after removing duplicate and unidentified species (Table 2). For a detailed list of species, see Supplmentary Table S1.

**Figure 2:**
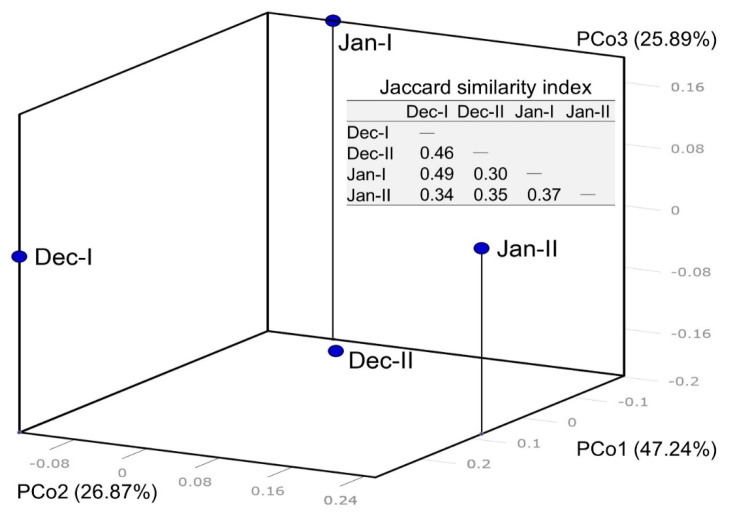
β-diversity patterns of fish species from 4 eDNA samplings (December-I, December-I, January-I, January-II) as inferred from Principal Coordinate Analysis (PCoA). The similarity matrix was obtained by the Jaccard similarity index.

In the four samplings, 81, 56, 126, and 60 species were obtained, respectively. There are 64, 52, and 15 species (12S, COI, ND5) in sampling December-I; 39, 44, and 11 species in December-II; 103, 68, and 17 species in January-I; 44, 52, and 14 species in January-II (Table 2). β-diversity patterns inferred from Principal Coordinate Analysis (PCoA) further showed the differences on fish species between the sampling dates (December and January) and sampling sites (I and II) (Figure 2). The pairwise similarity of each sample is between 0.3–0.49. The highest similarity is December-I and January-I, and the lowest similarity is December-II and January-I.

### 3.2. Seafood and Its Species Composition

These 153 species were further taxonomically classified into 2 classes (Actinopterygii and Chondrichthyes), 15 orders (Anguilliformes, Beloniformes, Clupeiformes, Gadiformes, Mugiliformes, Ophidiiformes, Pleuronectiformes, Salmoniformes, Scorpaeniformes, Siluriformes, Tetraodontiformes, Perciformes, Carcharhiniformes, Lamniformes, and Myliobatiformes), and 49 family, respectively (Table 3).

Chondrichthyan species are the major source of smoked sharks in fish market (Figure 1F). In the present study, a total of 22 chondrichthyan species were identified and taxonomically classified into to three orders (Carcharhiniformes, Lamniformes, and Myliobatiformes) and six families (Carcharhinidae, Sphyrnidae, Triakidae, Alopiidae, Dasyatidae, Urolophidae) (Table 4). Among these species, seven species were identified by both 12S and COI. Six and nine species were identified by 12S and COI, respectively (Table 4). Additionally, five species were consistently detected in all four samplings, including *Carcharhinus sealei*, *Sphyrna zygaena*, *Alopias pelagicus*, *Alopias superciliosus*, and *Maculabatis gerrardi* (Table 4). On the other hand, six species were only recorded in one sampling alone (Table 4). According to the IUCN Red List of Threatened Species (https://www.iucnredlist.org/ accessed on 15 August 2021), 17 species are threatened species, including two critically endangered (CR), eight endangered (EN), and seven vulnerable (VU) species (Table 4). Five species are near threatened (NT) (Table 4).

Anguilliformes species are the major source of braised moray eels (Figure 1G). In the present study, a total of 14 Anguilliformes species were identified and classified into two families, Congridae and Muraenidae (Table 5). Among these species, three species were identified by both 12S and COI. Thirteen and five species were identified by 12S and COI, respectively (Table 5). Additionally, two species were consistently detected in all four samplings, including *Gymnothorax flavimarginatus* and *Strophidon sathete* (Table 5). On the other hand, six species were only recorded once (Table 5). According to the species status of Taiwan Fishbase (http://fishdb.sinica.edu.tw/ accessed 25 August 2021), seven species are economic (E), three species are underused (U), three species are rare (R), and one species is endemic species of Taiwan (T) (Table 5).

Serranidae species (groupers) are economically important fish species and often used for grouper fish soups (Figure 1H). In the present study, a total of 12 Serranidae species were identified and classified into four genera, including *Aethaloperca*, *Cephalopholis*, *Epinephelus*, and *Variola* (Table 6). Among these species, four species were identified by both 12S and COI. Twelve and four species were identified by 12S and COI, respectively (Table 6). Additionally, *Epinephelus fasciatomaculosus* was the only species consistently detected in all four samplings. On the other hand, 4 species were recorder once (Table 6). Furthermore, seven groupers are wild species (W), and five groupers are commonly culture species in Taiwan (C) (Table 6).

## 4. Discussion

In the present study, we have demonstrated a novel approach for a large scale of seafood authentication using eDNA metabarcoding. Compared to the conventional DNA-based approach, it does not require a considerable number of DNA samples from seafood visible and accessible in the fish markets and/or seafood restaurants. Furthermore, it could also allow the simultaneous detection of multiple species. Despite the disruption of COVID19 pandemic for this work, we have still identified 153 fish species based on a limited number of eDNA samplings in a local fish market and its nearby seafood restaurants. Thus, we have shown the first evidence that it is feasible to provide a more comprehensive understanding of fish species potentially consumed in fish markets and restaurants using this novel approach.

The number of fish species identified from eDNA samplings in the present study were found to vary with different molecular markers. A total of 153 species were identified from eDNA samples in the present study, 112 species were identified by 12S and the rest of fish species were identified using COI and ND5. Indeed, 12S have proved to be the effective molecular marker for the detection of fish species from eDNA samples [25,34] and have been extensively utilized for the study of fish biodiversity using eDNA metabarcoding [36,37,38,39]. However, some studies also have shown the limitation of 12S for the detection of fish species from eDNA samples [40,41,42]. To maximize the number of fish species detected from eDNA samples in the present study, we have selected 19 universal primers targeted for three mitochondrial gene fragments (12S, ND5, COI). Additionally, the amplicons of these gene fragments from eDNA samples were further processed with relatively higher sequencing depth (6,995,762–11,021,896 reads per sample) in the present study. Inappropriate sequencing depth of eDNA samples might fail to detect those species with low abundance in the environments [43].

The number of fish species were also found to vary with sampling dates and sites in the present study. The differences in the number of fish species between different sampling dates might be explained by the availability of fish species since fishing seasons varied with fish species in Taiwan [23]. As for the differences in the number of fish species between two sampling sites in the present study might be explained by the nature of eDNA samples sourced from fish market and seafood restaurants. In the fish market, most fish species were still alive and/or unprocessed whereas most fish species in seafood restaurants are highly processed. The concentration of eDNA decays over time and its degradation rate also varies with different environmental conditions, including salinity, temperature, and pH [44].

Accumulatively, Chondrichthyan, Anguilliformes, and Serranidae species accounted for approximately one third of fish species identified from the fish market and its nearby seafood restaurants in the present study. These species are routinely used for the traditional seafood in Taiwan, including smoked sharks, braised moray eels and grouper soups (Figure 2F–H). Chondrichthyan species (sharks, rays, and chimeras) have continuously received much attention due to their declined abundance [45] and vulnerable life history traits (e.g., slow growth rates and long generation time) [46]. DNA barcoding analysis of Chondrichthyan species have revealed approximately 20 to 24 species involved in the seafood consumption in Taiwan [10,47]. In the present study, we have identified a total of 22 chondrichthyan species based on eDNA samplings from a limited area compared to previous studies in Taiwan [10,47]. It also worth mentioning that 17 species are now considered as IUCN threatened species. Anguilliformes are a group of eel-like shape species comprising 13 families and 206 species in Taiwan [48]. In the present study, 14 species were identified in the present study. Of these species, three species are rarely found, and one species is endemic species of Taiwan. These findings also highlight the potential use of eDNA metabarcoding for the conservation and managements of Anguilliformes in the future studies. Serranidae species, especially groupers, are globally popular seafood species with relatively higher economical value and many of them have been supplied by the aquaculture [49]. In the present study, 12 Serranidae species were identified, including seven wild and five aquaculture species. Notably, there is a huge difference in the market price between the wild and farmed groupers in Taiwan. However, these species are often simply named as the grouper in the fish markets and seafood restaurants in Taiwan. Our findings further imply the Serranidae species from both capture fisheries and aquaculture were routinely consumed in the fish markets and seafood restaurants in Taiwan.

Over the past decades, the global marine biodiversity is declining rapidly due to climate change, habitat deconstruction, and overfishing [50]. Seafood consumption also plays a crucial role in the maintenance of marine biodiversity. Our findings have unveiled the underestimation of species diversity in seafood of Taiwan, especially those routinely consumed in fish markets and seafood restaurants. However, further improvements and optimizations of eDNA metabarcoding in fish markets and seafood restaurants would be required for the future studies, including the increase of sampling times and sites, the development and employment of molecular markers effective for the other seafood species (e.g., cephalopods, decapod crustaceans) to provide more comprehensive understandings of species diversity in seafood routinely consumed in fish markets and seafood restaurants.

## 5. Conclusions

This work represents the first attempt to examine fish species diversity in traditional seafood using eDNA metabarcoding approach. With the aid of multiple DNA markers and high throughput sequencing, a considerable number of fish species were detectable from limited eDNA samplings while further optimization of this approach remains for future studies. Nevertheless, eDNA metabarcoding could offer a cost-effective and non-invasive tool for providing a general profile of fish species potentially consumed in fish markets and restaurants. The identification of various fish species routinely used for traditional seafood further suggests a potential underestimation of the species diversity in traditional seafood and is crucial for the conservation and management of marine resources.

## Figures and Tables

**Table 1:** A list of 19 universal primers validated for the amplification of mitochondrial genes fragments (12S, ND5, COI).

Primer Sets	Sequence	Reference
12S		Miya et al. 2015 [25]
MiFish-U-F	GTCGGTAAAACTCGTGCCAGC	
MiFish-U-R	CATAGTGGGGTATCTAATCCCAGTTTG	
MiFish-E-F	GTTGGTAAATCTCGTGCCAGC	
MiFish-E-R	CATAGTGGGGTATCTAATCCTAGTTTG	
ND5		Miya et al. 2015 [25]
MiFish-tuna-ND5-F	ATGTCCTTCCTCCTTATCGGCTG	
MiFish-tuna-ND5-R	TTGCCAGTGGCAGCTACGATC	
COI		Shokralla et al. 2015 [26]
Fish-miniA-F	ACIAAICAIAAAGAYATYGGC	
Fish-miniA-R	AARAAAATYATAACRAAIGCRTGIGC	
Fish-miniC-F	ACYAAICAYAAAGAYATIGGCAC	
Fish-miniC-R	GAARATCATAATGAAGGCATGIGC	
Fish-miniE-F	ACYAAICAYAAAGAYATIGGCAC	
Fish-miniE-R	CTTATRTTRTTTATICGIGGRAAIGC	
FishF1	TCAACCAACCACAAAGACATTGGCAC	Becker et al. 2011 [27]
FishF2	TCGACTAATCATAAAGATATCGGCAC	
FishR1	TAGACTTCTGGGTGGCCAAAGAATCA	
FishR2	ACTTCAGGGTGACCGAAGAATCAGAA	
Shark-COI-MINI-R	AAGATTACAAAAGCGTGGGC	Zahn et al. 2020 [28]
Shark-MiniV1-R	AAGATTATTACAAAAGCRTGRGC	
Shark-MiniV2-R	AAGATTATTACRAADGCRTGRGC	

**Table 2:** Summaries for the number of fish species based on the sequencing reads of 12S, COI, ND5 in 4 eDNA samples collected from two different dates and sites.

Items	Reads	12S	COI	ND5	All
December-I	6,995,762	64	52	15	81 *
December-II	10,194,137	39	44	11	56 *
January-I	22,323,200	103	68	17	126 *
January-II	11,021,896	44	52	14	60 *
All	50,534,995	112 *	88 *	18 *	153 *

*: duplicate species were omitted.

**Table 3:** Taxonomic classification of 153 fish species identified from eDNA sampling in Heping island fish market and its nearby seafood restaurants.

Class	Order	Family	Species	Class	Order	Family	Species
Actinopterygii				Actinopterygii			
	Anguilliformes	Congridae	2		Perciformes	Haemulidae	2
		Muraenidae	12			Istiophoridae	5
	Beloniformes	Scomberesocidae	1			Kyphosidae	1
	Clupeiformes	Engraulidae	1			Labridae	2
	Gadiformes	Gadidae	3			Lutjanidae	8
	Mugiliformes	Mugilidae	4			Malacanthidae	1
	Ophidiiformes	Ophidiidae	1			Mullidae	2
	Pleuronectiformes	Cynoglossidae	1			Nemipteridae	1
		Paralichthyidae	1			Nomeidae	1
	Salmoniformes	Salmonidae	1			Pempheridae	1
	Scorpaeniformes	Scorpaenidae	2			Pomacentridae	7
	Siluriformes	Loricariidae	2			Scombridae	12
	Tetraodontiformes	Diodontidae	1			Serranidae	12
		Monacanthidae	3			Siganidae	1
		Tetraodontidae	1			Sparidae	3
	Perciformes	Acropomatidae	2			Stromateidae	3
		Apogonidae	2			Trichiuridae	5
		Bramidae	1			Xiphiidae	1
		Caesionidae	3	Chondrichthyes			
		Carangidae	11		Carcharhiniformes	Carcharhinidae	7
		Centrolophidae	3			Sphyrnidae	2
		Channichthyidae	1			Triakidae	5
		Coryphaenidae	1		Lamniformes	Alopiidae	2
		Emmelichthyidae	1		Myliobatiformes	Dasyatidae	5
		Gempylidae	2			Urolophidae	1

**Table 4:** List of chondrichthyan species detected in this study.

Order	Family	Scientific Name	12S	COI	December-I	December-II	January-I	January-II	Status
Carcharhiniformes	Carcharhinidae	*Carcharhinus brevipinna*	1	0	0	1	0	1	VU
		*Carcharhinus falciformis*	1	0	0	1	0	0	VU
		*Carcharhinus macloti*	1	0	0	1	0	0	NT
		*Carcharhinus obscurus*	1	1	0	1	1	1	EN
		*Carcharhinus sealei*	0	1	1	1	1	1	NT
		*Carcharhinus sorrah*	1	0	0	1	0	0	NT
		*Prionace glauca*	1	1	0	1	1	1	NT
	Sphyrnidae	*Sphyrna lewini*	1	0	0	1	0	0	CR
		*Sphyrna zygaena*	1	1	1	1	1	1	VU
	Triakidae	*Galeorhinus galeus*	0	1	1	0	1	0	CR
		*Hemitriakis japanica*	0	1	1	0	1	0	EN
		*Mustelus asterias*	0	1	1	0	1	0	NT
		*Mustelus griseus*	0	1	1	1	0	0	EN
		*Mustelus manazo*	0	1	1	1	0	0	EN
Lamniformes	Alopiidae	*Alopias pelagicus*	1	1	1	1	1	1	EN
		*Alopias superciliosus*	1	1	1	1	1	1	VU
Myliobatiformes	Dasyatidae	*Himantura leoparda*	0	1	0	1	1	1	VU
		*Maculabatis gerrardi*	1	1	1	1	1	1	EN
		*Maculabatis pastinacoides*	1	1	0	0	1	0	EN
		*Pastinachus gracilicaudus*	0	1	0	1	1	1	EN
		*Pateobatis jenkinsii*	0	1	0	0	1	1	VU
	Urolophidae	*Urolophus aurantiacus*	1	0	0	1	0	0	VU

Note: The absence and presence of species is denoted as 0 and 1, respectively. Green colors highlight the presence of species identified by different mitochondria genes (12S and COI). Yellow colors highlight the presence of species identified in different sampling dates (December and January) and sites (I and II). Red colors highlight the threatened species. CR: critically endangered species; EN: endangered species; VU: vulnerable species; NT: near threatened species.

**Table 5:** List of Anguilliformes detected species in this study.

Order	Family	Scientific Name	12S	COI	December-I	December-II	January-I	January-II	Status
Anguilliformes	Congridae	*Bathycongrus retrotinctus*	1	0	0	1	0	0	R
		*Gnathophis nystromi*	0	1	0	1	0	1	R
	Muraenidae	*Gymnothorax flavimarginatus*	1	1	1	1	1	1	E
		*Gymnothorax isingteena*	1	1	1	1	1	0	U
		*Gymnothorax javanicus*	1	0	1	0	0	0	E
		*Gymnothorax prionodon*	1	0	0	1	0	0	E
		*Gymnothorax margaritophorus*	1	0	0	1	0	0	E
		*Gymnothorax niphostigmus*	1	0	0	1	0	0	T
		*Gymnothorax reevesii*	1	1	1	1	1	0	E
		*Gymnothorax reticularis*	1	0	0	1	1	1	U
		*Gymnothorax thyrsoideus*	1	0	0	1	1	1	E
		*Gymnothorax undulatus*	1	1	1	1	1	0	E
		*Enchelycore anatina*	1	0	0	1	0	0	U
		*Strophidon sathete*	1	0	1	1	1	1	R

Note: Green colors highlight the presence of species identified by different mitochondria genes (12S and COI). Yellow colors highlight the presence of species identified in different sampling dates (December and January) and sites (I and II). Red colors highlight the economic species. E: economic species; U: underused species; R: rare species; T: endemic species of Taiwan.

**Table 6:** List of Serranidae species detected in this study.

Order	Family	Scientific Name	12S	COI	December-I	December-II	January-I	January-II	Status
Perciformes	Serranidae	*Aethaloperca rogaa*	1	1	1	1	1	0	W
		*Cephalopholis boenak*	1	0	1	1	1	0	W
		*Cephalopholis sexmaculata*	1	0	1	1	1	0	W
		*Epinephelus awoara*	1	1	1	0	0	1	W
		*Epinephelus coioides*	1	1	1	1	1	0	C
		*Epinephelus fasciatomaculosus*	1	0	0	1	0	0	W
		*Epinephelus fuscoguttatus*	1	1	1	1	1	1	C
		*Epinephelus lanceolatus*	1	0	0	1	0	0	C
		*Epinephelus bruneus*	1	0	1	1	0	1	C
		*Epinephelus quoyanus*	1	0	1	1	0	0	W
		*Epinephelus tukula*	1	0	0	1	0	0	C
		*Variola louti*	1	0	0	1	0	0	W

Note: Green colors highlight the presence of species identified by 12S and COI. Yellow colors highlight the presence of species identified in different sampling dates (December and January) and sites (I and II). Red colors highlight the wild species. W: Wild species; C; Culture species.

## Data Availability

The datasets presented in this study can be found in online repositories. The names of the repository/repositories and accession number(s) can be found below: NCBI (accession: SAMN22314222, SAMN22314446, SAMN22314507 and SAMN22314542).

## Supplementary Materials

The following are available at https://www.mdpi.com/article/10.3390/biology10111132/s1, Table S1: The complete list of species identified from eDNA samplings in the present study.
